# Combination therapy with low-frequency ultrasound irradiation and radiofrequency ablation as a synergistic treatment for pancreatic cancer

**DOI:** 10.1080/21655979.2021.1995581

**Published:** 2021-12-07

**Authors:** Huiyang Wang, Wenxiu Ding, Hongwei Shi, Haiwei Bao, Yuting Lu, Tian an Jiang

**Affiliations:** a Department of Ultrasound Medicine, The First Affiliated Hospital, Zhejiang University School of Medicine, Hangzhou, China; b Department of Ultrasound Medicine, The First Affiliated Hospital of Zhejiang Chinese Medical University, Hangzhou, China; c Zhejiang Provincial Key Laboratory of Pulsed Electric Field Technology for Medical Transformation, Hangzhou, China

**Keywords:** Low-frequency ultrasound-stimulated microbubbles, radiofrequency ablation, combined treatment, Panc02 cell, apoptosis, autophagy

## Abstract

We aim to evaluate the efficacies of combination therapy with low-frequency ultrasound-stimulated microbubbles (USMB) and radiofrequency ablation (RFA) on suppressing the proliferation of pancreatic cancer cell and treating Panc02 subcutaneous xenograft mice. The proliferation of HPDE6-C7 and Panc02 cells after the treatment of USMB and RFA alone or combination were evaluated by CCK-8 assay. Scratch test was performed to assess the cell migration capability. Panc02-bearing mice were received 14-day treatment of USMB and RFA alone or combination. Tumor size and survival rate were recorded once two days. The serum levels of immune-related factors and changes of apoptosis- and autophagy-related factors were detected by ELISA and western blotting methods. As a result, CKK-8 assays revealed significant inhibition on Panc02 cell proliferation in combination therapy with USMB and RFA relative to other groups (all *p* < 0.05). Strong synergistic effect of USMB combined with RFA was confirmed via the calculated combination index (CI) <0.4. In addition, combination therapy of USMB and RFA significantly inhibited the migration of Panc02 cells. Moreover, combined treatment remarkably inhibited the size and width of xenograft and improved the survival in Panc02-bearing mice. Furthermore, 14-day combination therapy of USMB and RFA in Panc02-bearing mice significantly facilitated the apoptosis and autophagy of tumor cells. In summary, combination therapy of USMB and RFA showed synergistic anti-tumor efficacies on Panc02 cells attributing to the promotion on apoptosis and autophagy in Panc02 subcutaneous xenograft mice.

## 1. Introduction

In recent years, the pancreatic cancer is one of the most common and lethal malignancies in the digestive tract with the continuously increased incidence worldwide [1–3]. The mortality rate of pancreatic cancer ranks fourth highest among various malignancies, and the 5-year survival is less than 5% [4,5]. However, the anatomical location of the pancreas is concealed, and pancreatic cancer lacks specific clinical symptoms [6]. Most pancreatic cancers are often diagnosed in the mid- and late-stage, so only 10% to 15% of patients have the chance of surgical resection [7,8]. Gemcitabine-based first-line chemotherapy regimen resulted in limited survival benefit, with an overall survival time of about 6 months [9,10]. The combination chemotherapy regimen FOLFIRINOX improved the overall survival to 11.1 months [11]. Nevertheless, the enhancement of toxic side effects remains a huge restriction to the efficacy of FOLFIRINOX [12]. In terms of targeted therapy, erlotinib was the only FDA approved molecular targeted drug for the first-line treatment of advanced pancreatic cancer [13]. Significant progress in the treatment of pancreatic cancer has not been achieved over the past 30 years, and pancreatic cancer remains the greatest challenge in the anti-cancer campaign in the 21st century.

Low-intensity ultrasound, as a safe and noninvasive treatment, has been widely used in cancer treatment [14,15]. As one of the important effects of ultrasound biology, ultrasound cavitation significantly inhibits tumor cell proliferation and formation mainly through the mechanical effects and cavitation effects produced by ultrasound [14,16]. Studies have shown that ultrasound microbubble contrast agents can significantly enhance the cavitation effect produced by low-frequency and reduce the cavitation threshold, indicating that low-frequency ultrasound-stimulated microbubbles (USMB) has a significant inhibitory effect on tumor cell growth [17]. In addition, it has been confirmed that free radicals and microacoustic flow viscous stress generated by USMB can cause cell membrane, cytoskeletal damage and DNA fragmentation, thereby promoting the apoptosis in a variety of tumor cells, indicating that USMB has important experimental and clinical value in suppressing tumors [17].

Recently, electromagnetic field technology has been widely used in the biological category. Radiofrequency ablation (RFA), as a new non-thermal ablation method for tumors, has achieved remarkable efficacy in the clinic [18]. Compared with the traditional form of cancer treatment, RFA has some unique advantages: quick, controllable, visible and non-thermal mechanism, which can be used for the treatment of tumors adjacent to vital organs and tissues [18]. In addition, RFA is widely used to treat cancers that cannot be surgically removed and is characterized by the fact that it can also shrink tumors and improve patient viability on the basis of inhibiting tumor growth [18]. In this study, we hypothesized that combination therapy of USMB and RFA may trigger beneficial effects on pancreatic cancer in human pancreatic cancer xenograft models. To prove such a hypothesis, we evaluated the effects of USMB combined with RFA on cell proliferation and migration of Panc02 cells. Moreover, we also assessed the therapeutic effects of USMB combined with RFA on tumor size and survival of pancreatic cancer xenograft mice. In addition, the potential anti-tumor mechanisms were also investigated. Our study demonstrated that USMB and RFA exert synergetic effect on inhibiting pancreatic cancer via promoting apoptosis and autophagy.

## 2. Materials and methods

### 2.1 Materials

Ultrasound contrast agent was sulfur hexafluoride microbubbles SonoVue which purchased from Bracco Company (Milan, Italy). RPMI 1640 culture medium, fetal bovine serum and 0.25% trypsin were purchased from Gibco Company (CA, United States). Small pulsed focused ultrasonic cavitation therapeutic apparatus was purchased from WELLD Company (Shenzhen, China). The primary antibodies of B-cell lymphoma-2 (BCL-2), BCL-2-Associated X (Bax), LC3 II, LC3 I, caspase-3, Beclin-1, survivin, CyclinD1, p62, ATG5 and β-actin as well as goat anti-mouse secondary antibodies were purchased from Abcam (Cambridge, England). TNF-α, IFN-γ, IL-2 and IL-6 ELISA kits were acquired from BOSTER Company (CA, United States). S-1500 radiofrequency ablation therapeutic apparatus was obtained from MedSphere (Shanghai, China). All other reagents were acquired from Abcam (Cambridge, England) except indicated.

### 2.2 Animals

SPF BALB/c nude mice (female, 4–6 weeks, weighing 15 ~ 16 g) were purchased from Hangzhou Medical College (Hangzhou, Zhejiang). All mice were housed and bred in the controlled environment of room temperature 25 ± 1°C and relative humidity 40%~60% with 4 mice/cage in accordance with institutional guidelines for animal welfare at Hangzhou Medical College. All animal experimental experiments were performed followed the guidelines for Institutional Animal Care and Use Committee, ZJCLA with approval code of ZJCLA-IACUC-20060020.

### 2.3 Cell strain and cell culture

The mouse pancreatic cancer cell (Panc02 cell, BNCC338034) and normal pancreatic ductal epithelial cell (HPDE6-C7 cell, BNCC338285) were purchased from Beijing Beina Chuanglian Institute of Biotechnology (Beijing, China). After the cells were revived, the old culture medium was aspirated, and the dish was washed twice with PBS. Add 0.25% trypsin in a 2 mL/100 mm dish and observe under a microscope. When the cells had just detached, most of the trypsin was sucked off. The culture dish was transferred to an incubator for digestion, and then taken out after 5 min. The dish was gently shaken, and the cells were blown to suspension, and the digestion was terminated by added Roswell ParkMemorialInstitute-1640 (RPMI-1640) + 10% fetal bovine serum. The cells were centrifuged at 1200 rpm for 5 min and the supernatant was discarded. Subsequently, 2 mL of culture medium was added, and the cells were blown to suspension. The suspended cells are dispensed into multiple culture flasks and incubated in an incubator. Repeat the above operations until the required number of cells for the experiment is reached.

### 2.4 Cell proliferation inhibition test

The 1 mL 1 × 10^6^ cell/mL of HPDE6-C7 or Panc02 (containing 0%, 1%, 2%, 5%, 10%, 15%, 20% or 30% SonoVue, respectively) were added to 1.5 mL round-bottom EP tube and place it upside down on the ultrasonic probe. Low-frequency ultrasound with an acoustic intensity of 0.45 W/cm2 and an ultrasonic pulse wave frequency of 1 MHz was used for continuous irradiation for 30 s. In addition, 1 mL 1 × 10^6^ cells/mL of HPDE6-C7 or Panc02 cells was added to each electroporation cup (electrode spacing 4 mm). Then, each cup was placed in the electrode slot and applied for the radiofrequency electric field (pulse width 0, 0.1, 0.2, 0.5, 1, 2, 4 or 6 μs, respectively). In addition, as described above, we also investigated the effect of combined treatment with low-frequency ultrasound at different microbubble concentrations (5%, 10% or 15%) and radiofrequency electric fields at different pulse widths (0.5, 1 or 2 μs) on the viability of Panc02 cells. Each group of treated cells was inoculated into a 96-well plate with 100 μL/well. The plates were precultured at 37°C in 5% CO_2_ for 24 h. 10 μL CCK-8 was added to each well and mixed thoroughly. After incubating the plate in the incubator for 4 h, the absorbance at 450 nm was measured. The interaction of USMB and RFA was estimated by introducing the combination index (CI) scores. CI values were calculated by Eq (CI = D_1_/D_m1_+ D_2_/D_m2_, where D_1_ and D_2_ are the doses of intervention 1 and intervention 2 in combination that produce a specific effect, while D_m1_ and D_m2_ are intervention concentrations at which the same effect achieved when dosed individually). CI values of <0.4, 0.4–0.8, and >0.8 indicate strong, moderate, and slight synergistic effect, respectively.

### 2.5 Scratch test

The migration of Panc02 cells after combined treatment of USMB and RFA was characterized by scratch assay. In brief, the cells were treated following the above CCK-8 assay cell pretreatment method. Cells treated with USMB alone, RFA alone or a combination of USMB and RFA were repeatedly seeded in six replicate wells of a 96-well plate and allowed to settle overnight. A 700–800 µm wound was subsequently made in each well with a 100 μL pipette tip and washed for three times with sterile PBS solution. Panc02 cells were observed and randomly photographed under an inverted fluorescence microscope at 0 and 12 h. Cell migration rate was analyzed and calculated using Image J software.

### 2.6 Establishment of Panc02 subcutaneous xenograft model

Due to the absence of thymus in BALB/c, T cells cannot differentiate normally, resulting in T cell immunodeficiency. Therefore, this breed of mice has no contact sensitivity and no transplant rejection and can be widely used for the construction of transplanted tumor mice. Xenografted tumor mice were constructed as previously described [19]. Briefly, 0.1 mL of Panc02 cells at a concentration of 2 × 10^7^/mL were subcutaneously injected into BALB/C mice via the right axilla. The whole process is completed in the clean bench. After the completion of injection, the tumor formation was continuously observed, and the long and short diameters of tumor tissues were measured daily with a vernier caliper. The transplanted tumor volume V (cm^3^) was calculated as: V = (tumor width^2^ × tumor length)/2 [20]. The model with the tumor volume ≈ 100 mm^3^ was considered successfully established.

Forty Panc02-bearing mice were randomly divided into negative control group, USMB alone group (15% SonoVus), RFA alone group (2 μs pulse width), and combination group (USMB with 15% SonoVus and RFA with 2 μs pulse width. The tumor site was treated by low-frequency ultrasound after infusion of contrast agent, followed immediately by insertion of an electrode needle at the tumor site for tumor tissue ablation). Tumor sizes and survival rates of Panc02-bearing mice were recorded once two days. After 14 days of continuous treatment, peripheral blood was collected from mice by tail amputation, and the numbers of peripheral white blood cells, neutrophils, and monocytes were analyzed with LH-750 hematology analyzer (Beckman Coulter, USA). Meanwhile, the serum levels of Interferon-γ (IFN-γ), tumor necrosis factor-α (TNF-α), Interleukin-2 (IL-2), and Interleukin-6 (IL-6) were detected by specific ELISA kits. At the end of the treatment, mice were all euthanized and tumor tissues were removed and weighed. Tumor tissues were made to 5 μm sections after fixed in 10% formalin and embedded in paraffin for hematoxylin and eosin (H&E) staining.

### 2.7 Western blot analysis

Cell lysates and tissue homogenates were prepared by using chilled RIPA cell lysis buffer (containing protease inhibitors 50 × 20 μL) with gentle agitation. After high-speed hypothermal centrifugation, the supernatant was collected and the total protein concentration was determined by BCA kit. Supernatant protein samples were separated by 12.5% SDS-PAGE and electro-transferred to PVDF membranes. Subsequently, the membranes were sequentially blocked by TBST (containing 5% skim milk), and co-incubated by primary and secondary antibodies. Representative immunoblottings of protein were visualized by a chemiluminescence kit (Amersham Biosciences, USA).

### 2.8 Statistical methods

Statistical analysis was performed by One- or two-way ANOVA using Graphpad prism 8.0 software, and data were shown as Mean ± SD. *p* < 0.05 was designated as statistically significant difference.

## 3. Results

To investigate the anti-tumor effect and potential mechanism of USMB and RFA combination therapy on pancreatic cancer, we evaluated the effects of USMB combined with RFA on cell proliferation and migration of Panc02 cells. Moreover, we also assessed the therapeutic effects of USMB combined with RFA on tumor size and survival of pancreatic cancer xenograft mice. In addition, the potential anti-tumor mechanisms were also investigated.

### 3.1 Combination of USMB and RFA exhibited synergistic inhibition on Panc02 cell proliferation

**Figure 1.**
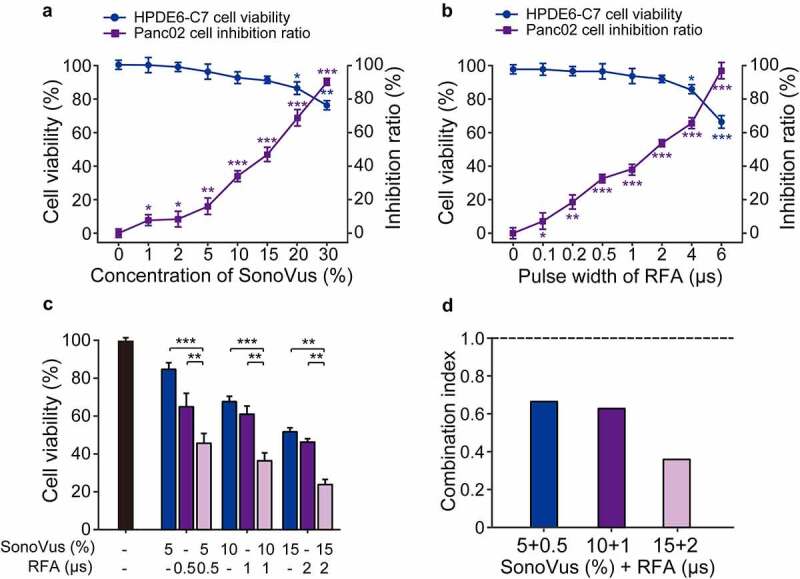
Activity of USMB and RFA combination against in vitro Panc02 cell proliferation. Effect of (a) USMB alone and (b) RFA alone treatment for HPDE6-C7 cell viability and Panc02 cell proliferation. (c) Effect of USMB and RFA combination on Panc02 cell viability and (d) the calculated CI values of USMB combined with RFA. *p < 0.05, **p < 0.01, ***p < 0.001 using one-way ANOVA. All data were presented as mean ± SD (n = 6)

Firstly, the in vitro cytotoxicity of USMB and RFA was assessed by CCK-8 assay. As shown in Figure 1, no significant effect was observed on the viability of normal HPDE6-C7 cells after USMB treatment with the gradually increase microbubble concentration from 0% to 15%. In addition, the inhibition rate of Panc02 cells was progressively increased under low-frequency ultrasound irradiation from 0% to 30% microbubble concentration. On the other hand, RFA with a pulse width of less than 2 μs significantly inhibited the proliferation of panc02 cells, but had no significant effect on normal HPDE6-C7 cell viability. Among them, the IC50 value was about 16.9 ± 0.9% for microbubble concentration of USMB, and about 1.81 ± 0.23 μs for pulse width of RFA. Subsequently, we investigated the effect of USMB combined with RFA on the viability of Panc02 cells. As the results showed in Figure 1, the USMB+RFA significantly inhibited Panc02 cell viability compared with the USMB- or RFA-alone treated groups (both *p* < 0.01). Moreover, the CI of the combined treatment of USMB with different microbubble concentrations and RFA with different pulse widths was less than 1.0. In particular, the combined treatment of USMB at 15% microbubble concentration and RFA at 2 μs pulse width showed a very strong synergistic effect (CI value <0.4).

### 3.2 Combination of USMB and RFA inhibited the migration of Panc02 cells

**Figure 2.**
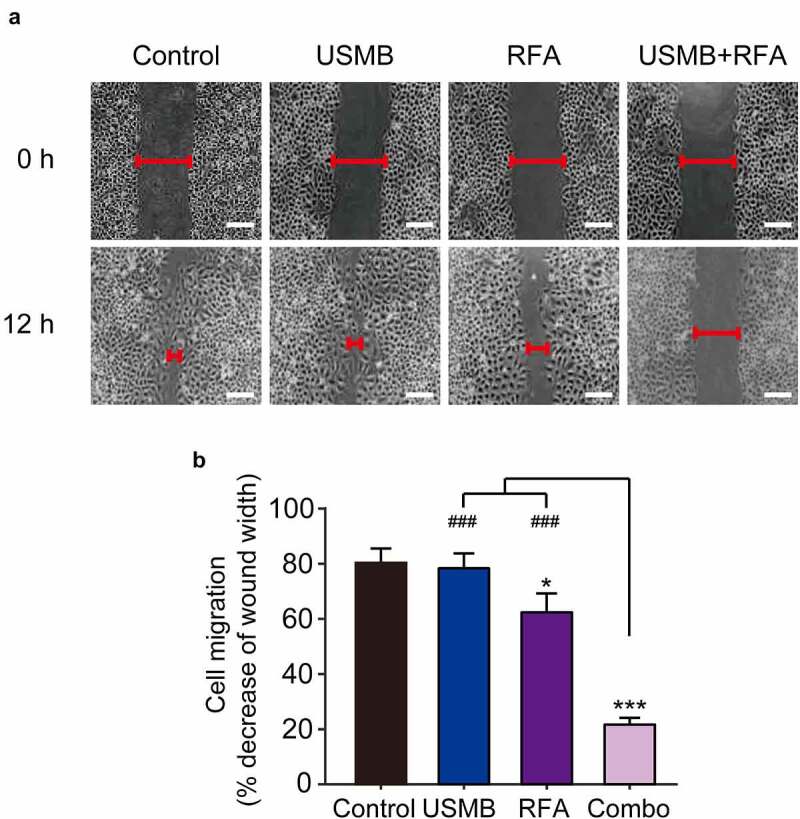
Effects of USMB and RFA combination therapy on inhibiting Panc02 cell migration (Scale bar = 100 μm). (a) The image and (b) decrease of wound width of Panc02 cell migration. *p < 0.05, **p < 0.01 and ***p < 0.001 vs. Control group; ^###^*p* < 0.001 vs. Combo group. Scale bar, 100 μm. All data were presented as mean ± SD (n = 6)

Scratch tests were performed to assess the combined effects on the in vitro migration ability of Panc02 cells. As shown in Figure 2, Panc02 cells in control group showed about 80% mobility after 12 hours. In contrast, USMB treatment alone slightly decreased the migration of Panc02 cells, but there was no significant difference. Interestingly, Panc02 cells following 12 h of combined treatment of USMB and RFA showed only about 22% migration, whereas ~60% migration was observed in Panc02 cells treated with RFA alone. These results collectively indicated a favorable cooperativity between USMB and RFA on inhibiting the migration of Panc02 cells.

### 3.3 Combination of USMB and RFA up-regulated expression levels of pro-apoptotic and autophagy-related proteins in Panc02 cell

**Figure 3.**
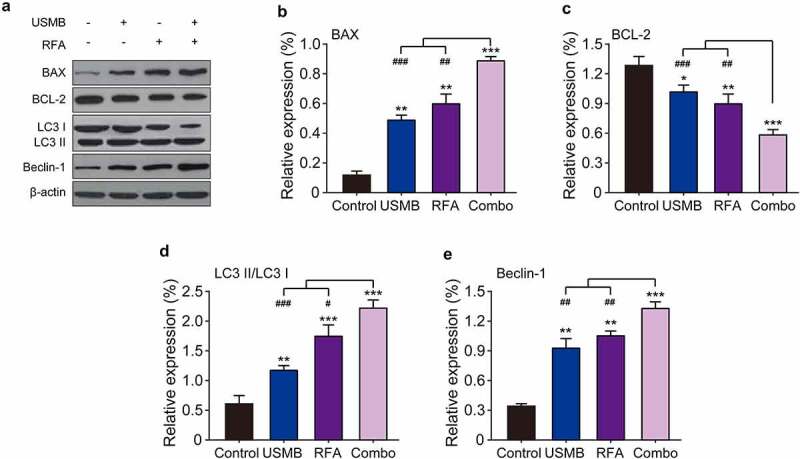
Western blot analysis of the effects of combination therapy of USMB and RFA on apoptosis- and autophagy-related proteins in Panc02 cells. (a) Representative western blot image and protein expression level of (b) BAX, (c) BCL-2, (d) LC3 II/LC3 I, and (e) Beclin-1. *p < 0.05, **p < 0.01 and ***p < 0.001 vs. Control group; ^#^*p* < 0.05, ^##^*p* < 0.01 and ^###^*p* < 0.001 vs. Combo group. Results were showed as means ± SD (n = 6)

To determine whether the anti-proliferative effect of the combined treatment of USMB and RFA resulted from apoptosis and autophagy, western blotting was used to measure apoptosis and autophagy-related proteins. As shown in Figure 3A–C, USMB treatment at a concentration of 15% microbubbles for 24 h significantly up-regulated the expression levels of BAX, a pro-apoptotic factor and BCL-2, an anti-apoptotic factor, respectively (both *p* < 0.05). Similar results were obtained in RFA alone treated group (*p* < 0.01). In addition, USMB combined RFA significantly enhanced the regulatory effects on the above apoptosis-related proteins (all *p* < 0.001). Similarly, the expression of autophagy-related proteins (LC3 I, LC3 II as well as Beclin1) in Panc02 cells were also examined. The results in Figure 3D showed that the combined treatment significantly promoted the conversion of LC3 I to LC3 II compared with USMB or RFA alone and the control group, as evidence by an increased proportion of LC3 II/LC3 I, indicating a feature of enhanced autophagy in Panc02 cells. In addition, Beclin-1 expression was also significantly enhanced in the combined treatment group (*p* < 0.001). All these results collectively demonstrated that the combined treatment of USMB and RFA could effectively promote autophagy as well as apoptosis of Panc02 cells and ultimately inhibited the tumor cell proliferation.

### 3.4 Combination of USMB and RFA effectively inhibited the pancreatic cancer growth in xenograft model

**Figure 4.**
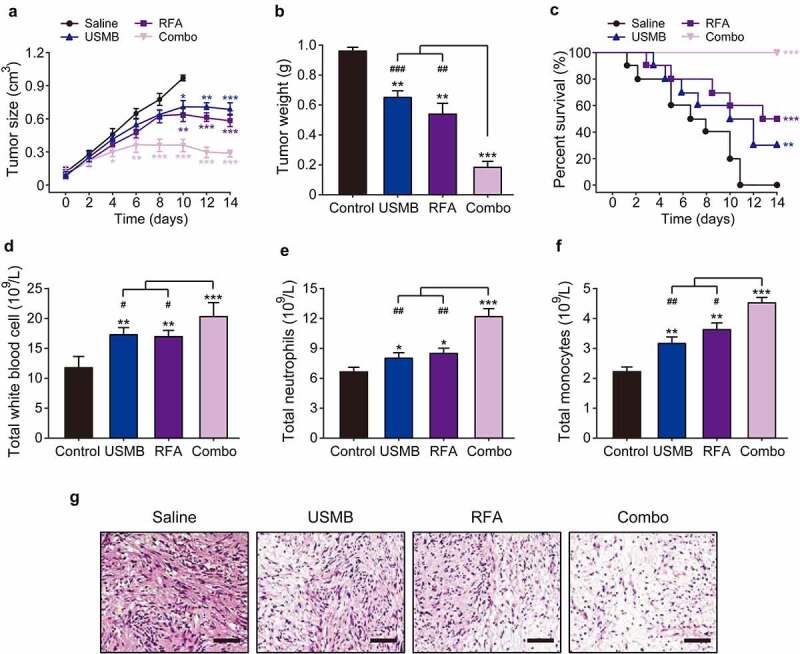
Effects of 14-day combination therapy of USMB and RFA on pancreatic xenograft in Panc02-bearing mice. (a) Tumor size, (b) tumor weight and (c) survival rate of Panc02-bearing mice; (d) Total white blood cells, (e) total neutrophils and (f) total monocytes in the peripheral blood of Panc02-bearing mice; (g) HE analysis of pancreatic carcinoma tissues of Panc02-bearing mice. *p < 0.05, **p < 0.01 and ***p < 0.001 vs. Control group; ^#^*p* < 0.05, ^##^*p* < 0.01 and ^###^*p* < 0.001 vs. Combo group. Scale bar, 100 μm. All data are expressed as mean ± SD (n = 10)

Considering the effective inhibition on the in vitro proliferation of Panc02 cells by combined treatment of USMB and RFA, the in vivo anti-tumor potentials of this combination therapy were further evaluated using Panc02 bearing mice. The mice were divided into four groups and, three of which treated with USMB, RFA, and the combination of USMB with RFA for 14 consecutive days, and the remaining group served as the negative control group. No significant muscle contraction, intolerance or on-site death were observed in Panc02 bearing mice during treatment. As shown in Figure 4A–B, the tumor volume in control mice continuously increase during 14-day treatment, while treatment with USMB or RFA alone significantly decreased the tumor volume and tumor weight of mice. Surprisingly, after 14 consecutive days of combined treatment with USMB and RFA, transplanted tumors in mice were almost completely eliminated without subsequent tumor recurrence. The xenograft mice in control group started to die two days after the start of treatment, and all of them were dead within 11 days (Figure 4C). Although treatment with USMB or RFA alone significantly improved the survival of mice, only three mice survived in the USMB-treated group, while only five survived in the RFA-treated ones. Interestingly, 100% survival rate of mice was observed in the combined treatment group, indicating that the combined treatment of USMB and RFA was effective in prolonging the survival of xenograft mice.

Leukocytes, neutrophils and monocytes, as the main effector cells of nonspecific immunity, play a very important role in the progression of tumors. At the end of treatment, mice were euthanized and blood samples as well as tumor tissues were collected. As shown in Figure 4D–F, the total number of leukocytes, neutrophils, and monocytes in blood were significantly enhanced in combination group, which was notably higher than those in the monotherapy or control groups. Additionally, the results in H&E staining showed that the tumor cells in the control group were very abundant but inconsistent in size and morphology, and unevenly arranged (Figure 4G). In contrast, the numbers of pancreatic cancer cells were significantly reduced in tumor-bearing mice treated with USMB combined RFA for 14 consecutive days, which were more pronounced than those in the monotherapy group. Above results demonstrated that combined treatment of USMB and RFA holds the potential to be developed as a promising novel antitumor therapy.

### 3.5 Combination of USMB and RFA effectively increased the serum levels of immune-related cytokines

**Figure 5.**
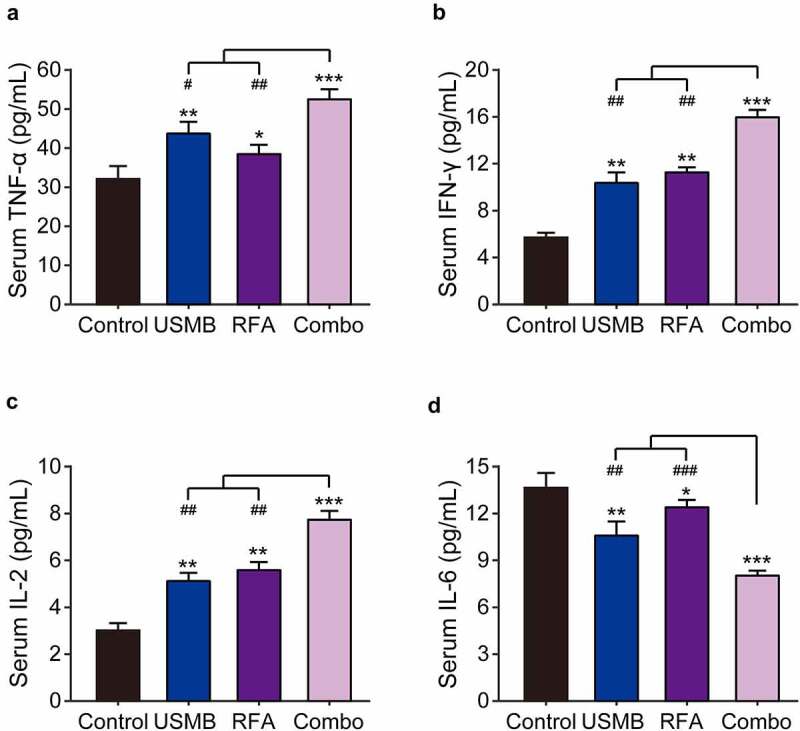
Effects of combination therapy of USMB and RFA on immune-related cytokines in Panc02-bearing mice. The serum levels of (a) TNF-α, (b) IFN-γ, (c) IL-2 and (d) IL-6 in Panc02-bearing mice. *p < 0.05, **p < 0.01 and ***p < 0.001 vs. Control group; ^#^*p* < 0.05, ^##^*p* < 0.01 and ^###^*p* < 0.001 vs. Combo group. Results were showed as means ± SD (n = 10 each group)

Imbalance of immune function is known to be a critical contributor in the pathogenesis of malignant tumors. Therefore, the serum levels of IFN-γ, TNF-α, IL-2, and IL-6 were examined via ELISA methods. As shown in Figure 5, USMB or RFA alone treatment significantly increased IFN-γ, TNF-α and IL-2 levels, and decreased IL-6 levels in peripheral serum of xenograft model compared with the control group. Moreover, the combination of USMB and RFA changes the levels of these cytokines to a greater extent compared to USMB or RFA alone treated group.

### 3.6 Combination of USMB and RFA enhanced apoptosis of Panc02 tumor cell in mice

**Figure 6.**
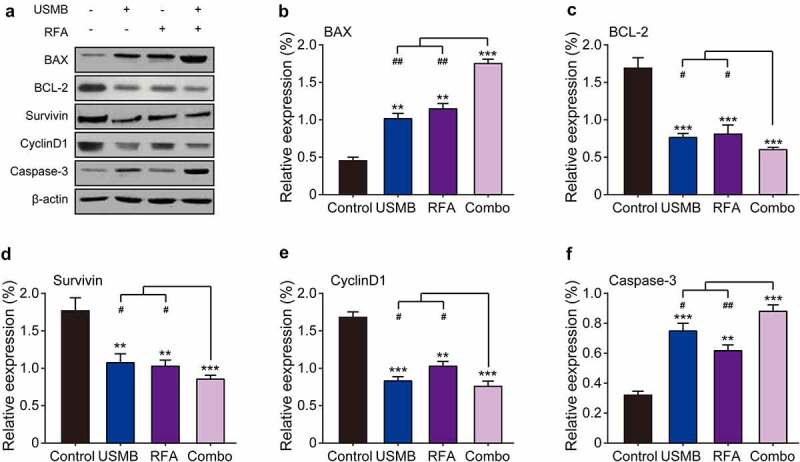
Western blot analysis of combination therapy of USMB and RFA on apoptosis-related proteins in Panc02-bearing mice. (a) Representative western blot image and protein expression level of (b) BAX, (c) BCL-2, (d) Survivin, (e) CyclinD1 and (f) Caspase-3 in pancreatic carcinoma tissues. *p < 0.05, **p < 0.01 and ***p < 0.001 vs. Control group; ^#^*p* < 0.05, ^##^*p* < 0.01 and ^###^*p* < 0.001 vs. Combo group. Results were showed as means ± SD (n = 10)

The expression of apoptosis-related proteins in tumor tissues of tumor-bearing mice was further investigated by western blotting. As showed in Figure 6, USMB or RFA alone significantly down-regulated the expression levels of Bcl-2, survivin and CyclinD1, while up-regulated those of Bax and caspase-3 compared with the control group. Furthermore, the USMB co-treated with RFA resulted in significantly higher reduction of Bcl-2, survivin and CyclinD1 expression and increase of Bax and caspase-3 expression compared to USMB or RFA treatment alone, suggesting that USMB synergizes with RFA to regulate the expression of the above apoptosis-related proteins and then jointly promote tumor cell apoptosis, inhibit cell proliferation, and finally inhibit the growth of subcutaneous xenografts of pancreatic cancer cell line Panc02.

### 3.7 Combination of USMB and RFA enhanced autophagy of Panc02 tumor cell in mice

**Figure 7.**
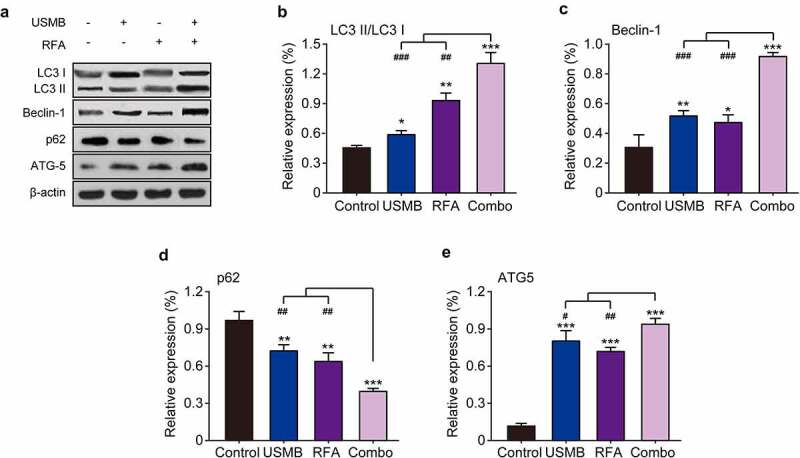
Effects of combination therapy of USMB and RFA on autophagy-related proteins in Panc02-bearing mice. (a) Representative western blot image and protein expression level of (b) LC3 II/LC3 I, (c) Beclin-1, (d) p62, and (e) ATG5 in pancreatic carcinoma tissues. *p < 0.05, **p < 0.01 and ***p < 0.001 vs. Control group; ^#^*p* < 0.05, ^##^*p* < 0.01 and ^###^*p* < 0.001 vs. Combo group. Results were showed as means ± SD (n = 10)

To investigate whether autophagy contributes to tumor cell death induced by USMB and RFA combination therapy in tumor-bearing mice, we measured the expression levels of relevant autophagy markers by western blotting. The results in Figure 7 showed that single treatment with USMB or RFA both enhanced LC3 II expression and decreased LC3 I expression, indicating increased conversion of LC3 I to LC3 II. In addition, the conversion of LC3 I to LC3 II was significantly enhanced by the combined treatment, which is consistent with the results of the in vitro cell assay. Moreover, two other autophagy-related proteins, Beclin-1 and ATG5, were significantly up-regulated after combined treatment with USMB and RFA. In contrast, p62, a marker protein of cellular autophagic activity, was significantly decreased after combined treatment with USMB and RFA. Above results collectively indicated that the combined treatment of USMB and RFA significantly enhanced the autophagy in tumor tissues from model mice, which further caused the development of apoptosis.

## 4. Discussion

Pancreatic cancer has an extremely invasive ability and easy to metastasize and relapse [1,2]. For early-stage pancreatic cancer, surgical resection has been considered as the preferred treatment [7]. However, no reliable method is available which allows for early diagnosis of pancreatic cancer, which results in low diagnosis rate [7]. The main treatment strategy for advanced pancreatic cancer is mainly systemic chemotherapy [9]. However, it is difficult for chemotherapeutic drugs to penetrate the dense and less fibrous capsule on the surface of pancreatic tumors, resulting in the insensitivity of most chemotherapeutic drugs [21]. In addition, acquired drug resistance in pancreatic cancer also manifests tumor recurrences growing [22]. To address the above conundrum, this study proposes a therapeutic approach of combining USMB and RFA for the treatment of pancreatic cancer and achieving ablation of solid tumors.

USMB, as a potential physical antitumor method, erodes tumor tissue by cavitation effect [14]. However, the process of tumor tissue ischemic necrosis induced by microvacuolization with ultrasound radiation is very slow and restricted, and the great vessels supplying the tumor are not completely destroyed [14]. RFA is a minimally invasive technique used for tumor tissue destruction by producing a resistive electrothermal effect through radio frequencies [23]. However, residual tumor cells may induce the localized recurrence [24]. We hypothesized that the combination of RFA and USMB treatment could use the destructive effect of RFA on tumor cells and the effect of ultrasound radiation microbubbles in the treatment of multiple tumor microvessels to perform tumor ultrasound radiation microbubbles treatment for the ablation zone and its periphery after RFA to make up for the lack of poor plasticity of RFA, that is, it can achieve the purpose of completely suitable treatment, reduce the destruction of normal tissues, maximize the protection of normal function, and further kill the residual tumor cells to achieve the best therapeutic effect, effectively ensuring the completeness of treatment. Hence, we explored the possibility of a combination treatment of USMB and RFA for pancreatic cancer.

Firstly, CCK-8 assay was used to investigate the effect of continuously increasing ultrasound microbubble concentration or radiofrequency pulse width on cell viability of HPDE6-C7 cells and the inhibition rate of Panc02 cells. The results showed that the microbubble concentration in the range of 1% ~ 15% or the radiofrequency pulse width in the range of 0.1 ~ 2 μs had no significant effect on the viability of HPDE6-C7 cells, but could significantly inhibit the proliferation of Panc02 cells. In addition, the combination of USMB and RFA inhibited the viability of Panc02 cells to a greater extent than the single treatment group. Of these, the combination of USMB at 15% microbubble concentration with RFA at radiofrequency pulse width of 2 μs showed the strongest synergistic inhibitory effect. Interestingly, studies have shown that USMB can inhibit the proliferation of tumor cells in vitro, but the apoptosis rate is very low and the anti-tumor effect is weak [25]. With the present of RFA, the in vitro anti-tumor effect of USMB was greatly improved, indicating that RFA has the potential to be an effective and additional anti-tumor supplementary treatment.

Invasiveness and metastasis are the most important biological features of malignant tumors [26]. Among them, cell migration motility is ubiquitous in the pathological process of tumor development [27]. Therefore, we investigated the effect of USMB and RFA combined therapy on Panc02 cell migration by scratch assay. The results demonstrated that the control Panc02 cells had produced significant migration at 12 h after scratching, while the USMB and RFA combination treatment group could significantly inhibit this migration, and the reduced migration distance was remarkably lower than the two single treatment groups, indicating that the synergistic treatment of USMB and RFA significantly inhibited the migration of Panc02 cells.

Both apoptosis and autophagy are modes of programmed cell death, meanwhile, are important factors that inhibit tumor cell proliferation and migration [28]. Autophagy is a lysosome-dependent degradation recycling process of cellular components involved in cell survival or cell death under a variety of adverse conditions [29]. Apoptosis is a process of rapid destruction of cell structure and organelles, resulting in cell shrinkage accompanied by nuclear chromatin condensation and nuclear fragmentation, ultimately leading to cell death [30]. Here, we investigated the relationship between inhibition of Panc02 cell proliferation and migration by combined treatment of USMB and RFA and apoptosis as well as autophagy. The results of Figure 3 showed that the combination treatment of USMB and RFA significantly down-regulated the BCL-2 and up-regulated BAX expression. Moreover, the expression levels of two autophagy-related factors, LC3 II and Beclin-1, were also significantly up-regulated in the combined treatment group. The above results suggested that the combination therapy of USMB and RFA may achieve the inhibition of proliferation and migration of Panc02 cells by regulating apoptosis and autophagy.

Given that USMB combined with RFA treatment had a strong inhibitory effect on the proliferation of Panc02 cells in vitro, we further investigated the in vivo antitumor effect in mice bearing subcutaneous human pancreatic cancer xenografts. We found that the growth of transplanted tumors in mice was significantly inhibited by USMB combined with RFA treatment, and the transplanted tumors in mice, surprisingly, were almost completely ablated at the end of treatment (Figure 4). At the same time, the survival of tumor-bearing mice was also greatly improved. Either pulsed electric field or low-frequency ultrasound irradiation with microbubbles can induce antitumor immune responses in vivo. As the main effector cells of nonspecific immunity, leukocytes, neutrophils and monocytes play a very critical role in the treatment of tumor. Enhancing the function of leukocytes and monocytes helps to destroy tumor cells and initiate the subsequent specific anti-tumor immune function, while the enhanced neutrophils can also exert nonspecific anti-tumor cell effects based on inflammatory feedback. As shown in Figures 4, 14-day treatment with USMB and RFA combination significantly increased the number of leukocytes, neutrophils, and monocytes in the peripheral blood of tumor-bearing mice, indicating that the combination therapy can induce in vivo nonspecific antitumor immune responses.

It is well known that the proliferation of cancer cells is closely related to apoptotic factors [31]. BAX and BCL-2 are pro- and anti-apoptotic factors, respectively [31]. Survivin is a member of the inhibitor of apoptosis protein family, which has the dual functions of inhibiting apoptosis and regulating cell division [30]. It is selectively expressed in a variety of common malignant tumors, but not in normal tissues and adult normal tissues. Caspase 3 is considered to be a key protease of mammalian apoptosis, a central molecule in the effector phase of apoptosis, and is involved in the physiological and pathological death process of cells [32]. Survivin inhibits the activity of Caspase 3 and blocks the apoptotic process of cells [32]. CyclinD1, on the other hand, belongs to the nuclear regulatory protein and is closely related to cancer cell proliferation [33]. CyclinD1 overexpression in cancer cells causes cell cycle shortening, which leads to excessive cell proliferation [33]. In this study, USMB combined with RFA treatment significantly down-regulated the expression of Bcl-2, survivin and CyclinD1, and up-regulated the expression of BAX and caspase-3 in xenograft of Panc02-bearing mice, indicating that the combination of USMB and RFA can inhibit the proliferation of tumor cells by promoting apoptosis (Figure 6). Surprisingly, a study of USMB in combination with RFA in mice with prostate xenografts revealed that incomplete thermal ablation of RFA has the potential to reduce apoptosis and stimulate proliferation of residual tumor cells [24]. While the treatment of USMB helped to reduce the extent of the proliferation-promoting effect in RFA, indicating that USMB treatment induced more cell lethal effects before the adaptive changes of the cells, which may also be the main cause of massive tumor cell apoptosis after the combination therapy of USMB and RFA.

Autophagy plays an important role in the occurrence and development of tumors, but the process of autophagy is strictly regulated by a series of proteins [29]. Among them, LC3 I and LC3 II are autophagy-relative markers [34]. Beclin-1 is a bridge between autophagy and apoptosis of tumor cell, and is also a key factor involved in the regulation of autophagosome formation, which is essential in the occurrence and development of tumors [35]. In addition, p62 can ubiquitinate target proteins and be degraded as degradation products encapsulate human autophagic vacuoles, which is an important marker of autophagy, that is, p62 can be degraded through autophagy, and autophagy is inhibited when its expression is enhanced [36]. ATG5 is a key factor involved in autophagosome formation and has various biological roles such as regulating autophagosome formation and inhibiting tumor generation [37]. After 14 days of USMB combined with RFA treatment in tumor-bearing mice, an increase in the conversion of LC3 I to LC3 II was observed, characterized by enhanced autophagy. Meanwhile, the combination of USMB and RFA also significantly up-regulated Beclin-1 and ATG5 and down-regulated the expression level of p62. The above results indicate that the combination of USMB and RFA can achieve anti-tumor effects by enhancing autophagy in tumor cells. The role of autophagy in the development and progression of tumors has not achieved widespread agreement [38]. We believe that autophagy is a mechanism to counteract cell carcinogenesis after cells exceed the low level of autophagic activity under normal conditions. As shown in the above results, the combination of USMB and RFA significantly promoted the autophagic death of tumor cells, thereby achieving a cancer-inhibiting effect.

## 5. Conclusion

In conclusion, the combined treatment of USMB and RFA showed a synergistic anti-pancreatic cancer effect in vitro and in vivo, and to a better extent than the monomeric treatment group. In addition, this study proposes that the combination of USMB and RFA achieves its anti-tumor effect by promoting apoptosis and autophagy of tumor cells, and the specific underlying mechanisms, unfortunately, have not been fully elucidated. One of the greatest novelties of our study is the combination of USMB and RFA to exert synergistic effect on inhibiting pancreatic cancer. We will deeply explore the specific potential mechanism of USMB and RFA combination in inhibiting pancreatic cancer in subsequent studies, and these findings will provide pharmacodynamics and theoretical basis for the future clinical use of this combination therapy strategy in the treatment of pancreatic cancer.

## Data Availability

All data generated or analyzed during this study are included in this article.

## Highlight

- USMB combined RFA inhibited the proliferation and migration of Panc02 cells
- USMB combined RFA suppressed the pancreatic cancer growth in xenograft model
- USMB combined RFA increased immune-related cytokines levels in xenograft model
- USMB combined RFA enhanced apoptosis and autophagy in Panc02 cells and xenografts
